# Dual network analysis of transcriptome data for discovery of new therapeutic targets in non-small cell lung cancer

**DOI:** 10.1038/s41388-023-02866-5

**Published:** 2023-10-20

**Authors:** Yuquan Bai, Lu Zhou, Chuanfen Zhang, Minzhang Guo, Liang Xia, Zhenying Tang, Yi Liu, Senyi Deng

**Affiliations:** 1 Institute of Thoracic Oncology and Department of Thoracic Surgery, West China Hospital, Sichuan University, Chengdu, 610041 China; 2 College of Computer Science, Sichuan University, Chengdu, 610041 China

**Keywords:** Non-small-cell lung cancer, Drug development

## Abstract

The drug therapy for non-small cell lung cancer (NSCLC) have always been issues of poisonous side effect, acquired drug resistance and narrow applicable population. In this study, we built a novel network analysis method (difference- correlation- enrichment- causality- node), which was based on the difference analysis, Spearman correlation network analysis, biological function analysis and Bayesian causality network analysis to discover new therapeutic target of NSCLC in the sequencing data of BEAS-2B and 7 NSCLC cell lines. Our results showed that, as a proteasome subunit coding gene in the central of cell cycle network, PSMD2 was associated with prognosis and was an independent prognostic factor for NSCLC patients. Knockout of PSMD2 inhibited the proliferation of NSCLC cells by inducing cell cycle arrest, and exhibited marked increase of cell cycle blocking protein p21, p27 and decrease of cell cycle driven protein CDK4, CDK6, CCND1 and CCNE1. IPA and molecular docking suggested bortezomib has stronger affinity to PSMD2 compared with reported targets PSMB1 and PSMB5. In vitro and In vivo experiments demonstrated the inhibitory effect of bortezomib in NSCLC with different driven mutations or with tyrosine kinase inhibitors resistance. Taken together, bortezomib could target PSMD2, PSMB1 and PSMB5 to inhibit the proteasome degradation of cell cycle check points, to block cell proliferation of NSCLC, which was potential optional drug for NSCLC patients.

## Introduction

With the development of clinical diagnosis and treatment, the 5-year postoperative survival rate of early NSCLC patients has been significantly improved [1]. Unfortunately, there are still a large number of NSCLC patients who were in the advanced stage, and needed adjuvant drug treatment to improve the prognosis after operation [2]. The existing clinical drug treatment of NSCLC mainly includes chemotherapy, small molecule targeted therapy and immunotherapy [3–8]. Chemotherapy is applicable to a wide range of patients, but the toxic and side effects are severe and the individual benefits of patients vary greatly [9]. Small molecule targeted therapy has strong pertinence and light adverse reactions because it directly acts on tumor driven signals such as EGFR, ALK, ROS1 and KRAS. It is the preferred way of drug treatment for NSCLC at present. However, the prevalence of drug resistance within 1–2 years after treatment makes it difficult to benefit for a long time in cancer patients [6]. Immunotherapy is considered as a new and reliable strategy for the treatment of NSCLC. Antibodies and small molecule drugs against immune check points such as PD-1, PD-L1 and CTLA-4 have been approved for clinical and achieved good therapeutic effects, but the low response rate limits the further improvement of its efficacy [10, 11]. Optimizing drug therapy and improving the therapeutic effect are the urgent problems in the clinical treatment of NSCLC nowadays.

Individualized precise treatment based on tumor pathology, cell biology and molecular biology has become the main development direction for clinical NSCLC treatment [12–14]. The establishment of effective drug efficacy evaluation system can match the optimal drug treatment for NSCLC patients, improve the treatment response rate, avoid unnecessary toxic and side effects and delay the occurrence of drug resistance. At the same time, the discovery of new targets and the development of new treatment strategies can also provide more treatment options for NSCLC patients, meeting the current needs of individualized and accurate treatment of NSCLC. The previous exploration of NSCLC targets focused on the structural variation or differential expression at the molecular level among different tissues or patients, such as the identification of EGFR mutation, KRAS mutation and ALK fusion in small molecule targeted therapy [15–17]. As we all know, tumor, as a persistent malignant tissue, has extremely complex pathological behavior and molecular regulation. It is the result of the participation of a variety of cells and the dynamic interaction of a large number of molecules [18]. It is difficult to find effective targets and tumor characteristics by simplifying this complex system from the perspective of molecular structure variation or expression difference.

Network science is regarded as a sharp weapon to analyze complex systems. The reason is that by abstracting system components and relationships into nodes and edges, complex systems can be transformed into mathematical network models. With the widespread application of omics detection technology in tumor research, it is easier to obtain the omics data of tumor DNA, RNA, protein, metabolites and other molecules. On the basis of considering the structural variation or differential expression, introducing correlation or causality network can better fit the internal characteristics of the complex system of tumor, and it has become a new path for the exploration of therapeutic targets [19–22].

In fact, network analysis has been widely used in disease research. In the enrichment analysis of tumor pathway, we usually rely on the correlation relationship between genes to annotate the function of target gene groups [23]. At the same time, network analysis is also used to describe the molecular features and dynamic progress of diseases [24, 25]. The network analysis of the existing tumor omics data is mostly based on the correlation between molecules. The limited conditions of the correlation between molecules in the network are weak and the network swing is large, which is not enough to accurately describe the internal characteristics of the tumor [26]. However, most physical and biological processes can be naturally modeled as causality networks [27]. Bayesian theorem is used to conduct causality analysis on tumor omics data. The causality molecular network constructed is more stable and accurate than the correlation molecular network.

We tried to integrate the existing methods of difference analysis, correlation analysis, functional enrichment and causality analysis to construct a new network analysis method of tumor transcriptome data, so as to discover the effective therapeutic targets. In this study, we built a new “difference-correlation-enrichment-causality-node” network construction and node capture technology process of tumor transcriptome data. Among them, “difference” refers to differential expression genes or differential network nodes, “correlation” refers to Pearson or Spearman correlation coefficient analysis, “enrichment” refers to functional enrichment analysis and “causality” refers to Bayesian probability analysis. This network analysis method is different from the previous network analysis methods. Based on the double test of correlation and causality, the molecular relationship described has higher authenticity and reliability. Relies on layer-by-layer dimensionality reduction and uses module segmentation and parallel operation, this method overcomes the problems of limited input data scale and slow operation speed of Bayesian probability calculation to a certain extent. Further, taking prognosis information as dependent variables and nodes as independent variables, we have accurately screened potential candidate genes by Cox regression analysis.

**Fig. 1:**
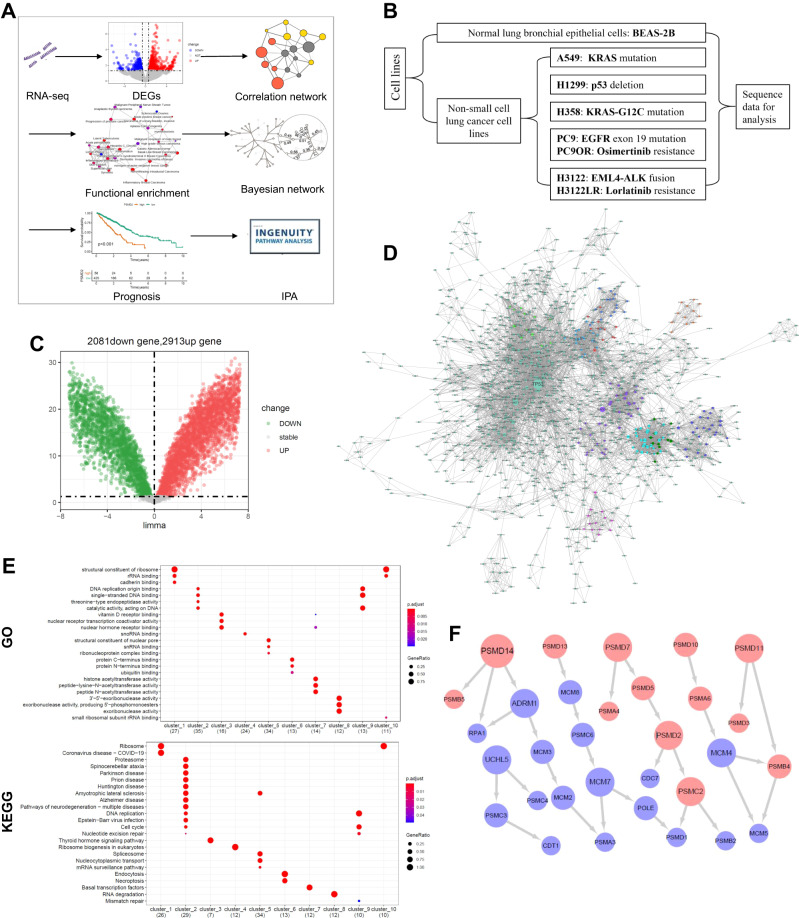
Identifies candidate target genes of NSCLC by transcriptome network analysis. **A** Flowchart of transcriptome network analysis. **B** Display of seven types of NSCLC cell lines and one type of normal bronchial epithelial cell line. **C** Volcano showed the differential genes between NSCLC cell lines and normal bronchial epithelial cell line. *P* < 0.05. **D** Correlation network analysis of differential genes. Different colors represent different clusters, and the node size represents the degree size of genes. **E** GO term and KEGG pathway analysis of ten functional clusters. **F** The causality network of cluster_2 and cluster_9. The red nodes represent proteasome-related genes, the node size represents the out-degree size of genes, and the arrow direction represents the causal relationship between genes.

In this study, we combined existing difference analysis, correlation analysis, functional enrichment and causality analysis to construct a transcriptome network analysis method for discovering new therapeutic target of NSCLC (Fig. 1A). Prognostic analysis, pathological examination, drug protein interaction analysis, molecular cell experiment and in vivo treatment assay were executed to investigate the function of selected gene and its potential as a novel therapeutic target of NSCLC.

## Results

### Proteasome subunit coding genes is located in the central of cell cycle regulation network

First, the sequencing gene sets of seven NSCLC cell lines and one normal bronchial epithelial cell line were intersected to obtain an expression matrix consisting of 9639 common genes and 23 cell samples (Fig. 1B). Then, batch correction (Fig. S1A–D) and difference analysis was performed, and 4994 differential expression genes were obtained (Fig. 1C). Next, correlation network analysis was carried out in these differential expression genes (Fig. 1D), then this correlation network was clustered by “MCODE” algorithm and yielded ten functional clusters (Fig. S2A–J and Table S1). After functional enrichment analysis of 10 clusters, we found that cluster_2 and cluster_9 were mainly enriched in cell cycle pathway and DNA replication (Fig. 1E). Therefore, we chose these genes from cluster_2 and cluster_9 for further analysis. We performed Bayesian causality network analysis on the new expression matrix of cluster_2 and cluster_9. According to weight >0.8 and out-degree ≥2, we finally identified 9 candidate genes (PSMD14, UCHL5, PSMD7, PSMD11, PSMC2, PSMD2, MCM7, MCM4 and ADRM1), which were mainly proteasome subunit coding genes (Fig. 1F and Table S2).

### PSMD2 is an independent prognostic factor for NSCLC

**Fig. 2:**
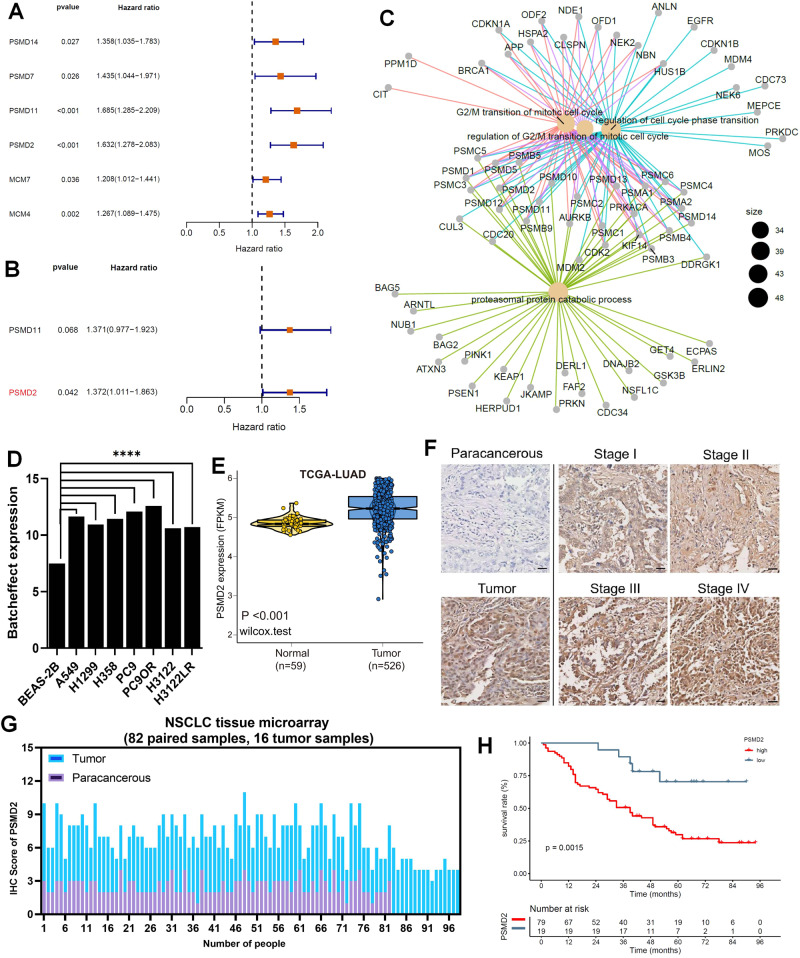
Identifies PSMD2 as an independent prognostic factor for NSCLC. **A** Univariate Cox regression analysis to screen the prognosis-related genes in NSCLC. **B** Multivariate Cox regression analysis identified gene with independent prognostic value in NSCLC. *P* < 0.05. **C** Functional enrichment of 269 PSMD2-related proteins. **D** Histogram showed the expression of PSMD2 in BEAS-2B and seven types of NSCLC cell lines. ****P < 0.0001. **E** Boxplot showed the expression of PSMD2 between tumor and normal in LUAD from TCGA database. **F** IHC staining showed the staining of PSMD2 in NSCLC patient tissues (scale bars = 20 μm). **G** Histogram showed the IHC scores of PSMD2 in 98 NSCLC patient tissues. **H** Grouping based on the IHC score of PSMD2, the survival curve was drawn for 98 NSCLC patients. In the IHC score, 0–3 scores were defined as low expression of PSMD2 in NSCLC tissues, and 4–7 scores were defined as high expression of PSMD2 in NSCLC tissues.

Based on univariate Cox regression analysis, we got 6 candidate genes (PSMD4, PSMD7, PSMD11, PSMD2, MCM7 and MCM4) were associated with poor prognosis of NSCLC patients in TCGA database (Fig. 2A and Fig. S3A). STRING network analysis identified the correlation network of 6 candidate genes (Fig. S3B). We found that the PSM family genes were a cluster, while the MCM family genes were a cluster. Moreover, both PSM and MCM family genes were associated with tumor progression [28, 29]. Then, these genes were simplified by lasso regression (Fig. S3C, D), and finally multivariate Cox regression analysis was performed with 2 genes (PSMD11 and PSMD2). The result showed that only PSMD2 could be served as an independent prognostic factor for NSCLC (*P* < 0.05, Fig. 2B and Table S3). And the PPI network analysis found that 269 PSMD2-related proteins were mainly enriched in cell cycle regulation and proteasome degradation (Fig. 2C).

Furthermore, we examined the expression of PSMD2 in NSCLC and its effect on the prognosis of NSCLC patients. Based on the TCGA-LUAD and our sequencing data, we found that PSMD2 was significantly higher expressed in NSCLC tissues and cells than in normal lung tissues and cells (*P* < 0.001, Fig. 2D, E). Meanwhile, we constructed three tyrosine kinase inhibitors (TKIs) resistance NSCLC cell lines (PC9OR and HCC827OR: osimertinib resistance; H3122LR: lorlatinib resistance) (Fig. S4A–C), and we found that PSMD2 was highly expressed in 9 types of NSCLC cells (Fig. S4D). And based on the tissue microarray composed of 98 NSCLC and 82 adjacent cancer samples (Table S4), we found stronger staining and higher IHC score of PSMD2 in NSCLC tissues than adjacent tissues (Fig. 2F, G). And the IHC score of PSMD2 was significantly correlated with tumor stage and lymph node invasion (*P* = 0.001, 0.014, Table 1). Further, based on univariate and multivariate Cox regression analysis of clinical characteristics in NSCLC patients, we found that tumor stage and lymph node invasion were independent prognostic factors, but PSMD2 score was not as limited by the few number of patients (*n* = 95) in the NSCLC tissue microarray (*P* = 0.034, 0.045, 0.053, Table S5). In addition, based on the prognosis information of 98 NSCLC patients, we found that the patients with high expression of PSMD2 (+) had a worse prognosis (*P* = 0.0015, Fig. 2H and Fig. S4E). Combining the above results, we identified that PSMD2 was associated with poor prognosis of NSCLC patients and was an independent prognostic factor for NSCLC.

**Table 1:** The correlation between PSMD2 score and clinical characteristics in NSCLC patients.

Clinical pathological parameters		PSMD2		*p* values
		−	+	
Gender	Male	8	49	0.075
	Female	11	27	
Age	<55	5	21	0.908
	≥55	14	55	
Tumor_size	<5	16	54	0.244
	≥5	3	22	
Tumor_stage	IA–IIB	18	42	0.001*
	IIIA–IV	1	34	
Lymph_node_examined_count	<12	12	50	0.829
	≥12	7	26	
Lymphatic_invasion	<1	14	32	0.014*
	≥1	5	44	

“−” represents the immunohistochemical score of PSMD2 ≤ 3, “+“ represents the immunohistochemical score of PSMD2 > 3.

*P < 0.05, marked in bold.

### Knockout of PSMD2 inhibits the proliferation of NSCLC cells by regulating protein degradation of cell cycle check points

**Fig. 3:**
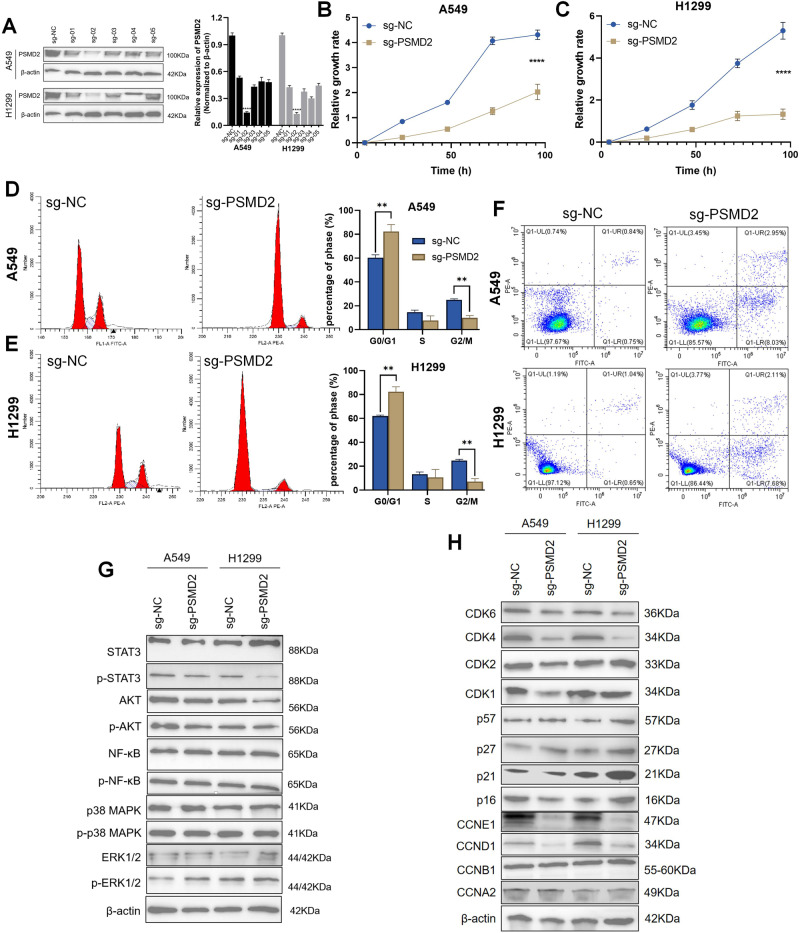
Knockout of PSMD2 inhibits the progression of NSCLC cells. **A** The gray value histogram of knockout efficiency of PSMD2 in A549 and H1299 cells. **B**, **C** Cell growth rates of A549-sgPSMD2 and H1299-sgPSMD2 groups after cell culture for 4, 24, 48, 72 and 96 h. ****P < 0.0001. **D**, **E** The percentages of A549-sgPSMD2 and H1299-sgPSMD2 groups at different phase (G0/G1, S and G2/M) of cell cycle were detected by flow cytometry. **P < 0.01. **F** Cell apoptosis of A549-sgPSMD2 and H1299-sgPSMD2 groups were detected by flow cytometry. **G** The phosphorylation protein expression of STAT3, NF-κB, PI3K-AKT, MAPK-ERK1/2 and MAPK-p38 pathways were detected in A549-sgPSMD2 and H1299-sgPSMD2 groups. **H** The expression of cell cycle pathway-related molecules was detected in A549-sgPSMD2 and H1299-sgPSMD2 groups.

To clarify the role of PSMD2 in the progression of NSCLC, we knocked out PSMD2 in A549 and H1299 cells, and the result showed that the knockout efficiency of sg-02 in both cells was close to 90% (Fig. 3A). Therefore, we chose sg-02 for further research. After knocked out PSMD2, we found that the growth rate of A549 and H1299 cells was slowed (*P* < 0.0001, Fig. 3B, C). In the G0/G1 phase of cell cycle, compared with the sgNC group, the mitosis of A549 and H1299 cells in the sg-PSMD2 group was blocked, and the DNA content was significantly increased (P < 0.01, Fig. 3D, E). Meanwhile, compared with A549-sgNC and H1299-sgNC groups, the cell apoptosis in the A549-sgPSMD2 and H1299-sgPSMD2 groups was significantly increased (*P* < 0.01, Fig. 3F).

Finally, we examined the expression of cascade signaling pathway and cell cycle pathway after knockout PSMD2 in NSCLC cells. We found that compared with the A549-sgNC and H1299-sgNC groups, the phosphorylated protein expression of STAT3, PI3K-AKT, NF-κB, MAPK-p38 and MAPK-ERK1/2 signaling pathways in the A549-sgPSMD2 and H1299-sgPSMD2 groups had no significant changes (Fig. 3G). But in the cell cycle pathway, compared with the A549-sgNC and H1299-sgNC groups, the expression of cell cycle blocking protein p21 and p27 had increased, and the expression of cell cycle driven protein CDK4, CDK6, CCND1 and CCNE1 had decreased in the A549-sgPSMD2 and H1299-sgPSMD2 groups (Fig. 3H).

### Bortezomib shows strong affinity to PSMD2

**Fig. 4:**
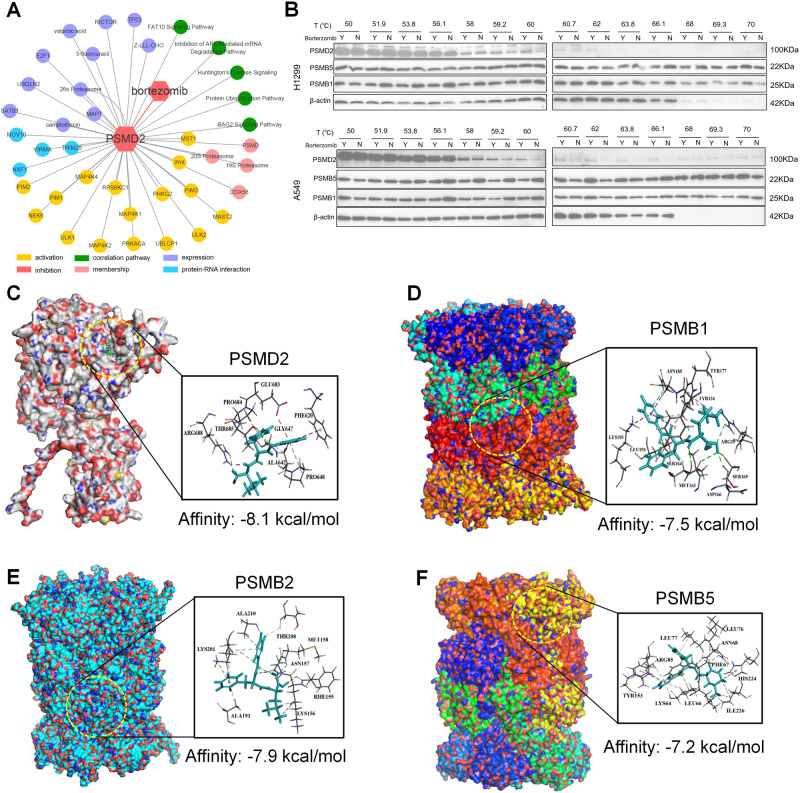
Bortezomib could target to PSMD2. **A** The network diagram shows the results of small molecule drug matching, correlation pathway analysis, protein-protein correlation analysis, protein-RNA correlation analysis and family member analysis of PSMD2. **B** The combination of bortezomib and PSMD2 was detected in H1299 and A549 cells with 14 temperature gradients set between 50 °C and 70 °C by thermal shift assay. Y represents cells were treated with bortezomib for 1 h, and N represents treatment with an equal amount of DMSO for 1 h. **C**–**F** Affinity of bortezomib with PSMD2, PSMB1, PSMB2 and PSMB5 were detected by molecular docking.

Since PSMD2 played an important role in the progression of NSCLC, we entered it into the IPA to analyze its biological functions and potential targeted drugs. We found that PSMD2 was matched to a selective inhibitor of 26S proteasome subunits, bortezomib (Fig. 4A). And most of activated proteins by PSMD2 were related with cell proliferation, which were also consistent with the result of functional enrichment of PSMD2 (Fig. 2C).

To verify the reliability of this result, we examined whether bortezomib could bind to PSMD2 by thermal shift assay. We found that in H1299 and A549 cells, after treated with bortezomib, from 59.2 °C to 62 °C, the expression of PSMD2 was significantly increased compared to the control group (Fig. 4B). This result indicated that bortezomib could bind to PSMD2 to form a stable complex, which was not easily decomposed during heating. We further refined the temperature gradient and found that in H1299 and A549 cells, the most stable complexes were formed at 60.2 °C (Fig. S5).

In addition, we performed molecular docking to verify the binding of bortezomib to PSMD2. As shown in Fig. 4C–F, the docking scores of the four complexes were all less than −5 kcal/mol, indicating that the corresponding compounds and targets have higher binding affinities. Among them, the docking score of bortezomib-PSMD2 was −8.1 kcal/mol, indicating a strong affinity as contrasted to reported bortezomib targets PSMB1 and PSMB5 [30, 31]. Based on these results, we determined that bortezomib have strong affinity to target PSMD2 in NSCLC.

### Bortezomib inhibits the proliferation of NSCLC cells by cell cycle arrest

**Fig. 5:**
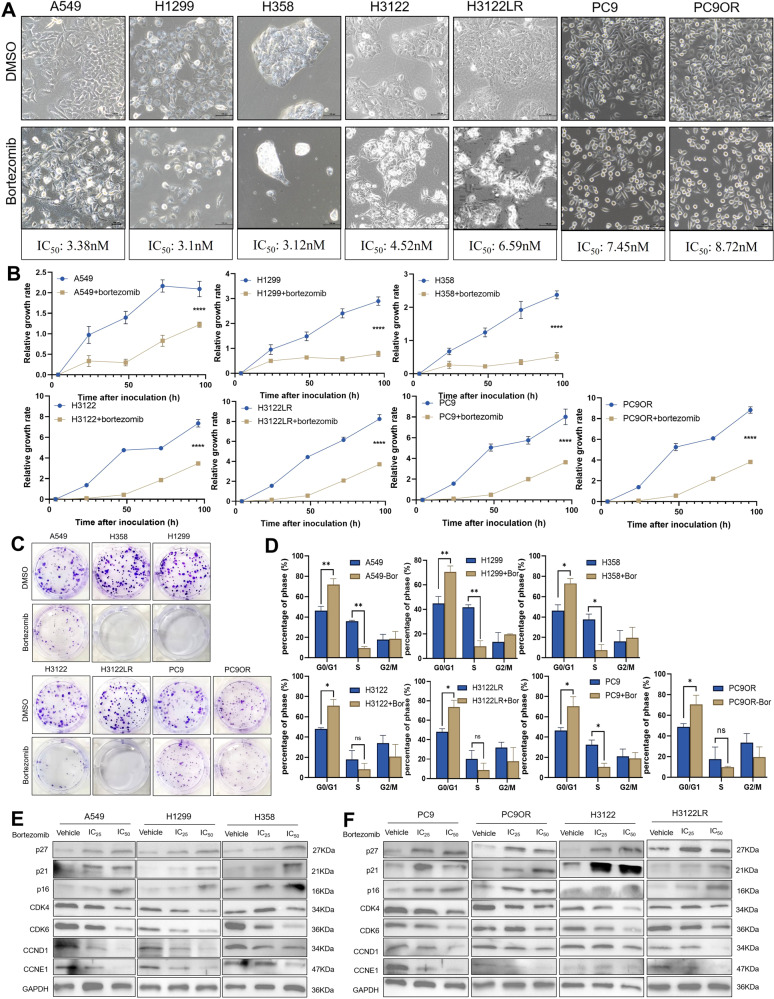
Bortezomib inhibits the proliferation of NSCLC cells by cell cycle arrest. **A** The picture showed the changes in cell morphology and number of different NSCLC cells treated with bortezomib (scale bar =100 μm). **B** The line chart showed the cell growth rates of bortezomib-treated group and control group cells in seven types of NSCLC cells. ****P < 0.0001. **C** The number of cell clones in the bortezomib-treated group and the corresponding control group was counted by clone formation experiments. **D** Histogram showed the percentages of seven types of NSCLC cells in different phase of cell cycle (G0/G1, S and G2/M) after treated with bortezomib. Bor: bortezomib, *P < 0.05, **P < 0.01. **E**, **F** The protein expression of cell cycle related molecules in A549, H1299, H358, PC9, PC9OR, H3122 and H3122LR cells under different concentrations of bortezomib. The IC_50_ of A549 was 3.38 nM and the IC_25_ was 2.88 nM; the IC_50_ of H1299 was 3.1 nM and the IC_25_ was 2.76 nM; the IC_50_ of H358 was 3.12 nM and the IC_25_ was 2.72 nM. The IC_50_ of PC9 was 7.45 nM and the IC_25_ was 5.83 nM; the IC_50_ of PC9OR was 8.72 nM and the IC_25_ was 6.24 nM; the IC_50_ of H3122 was 4.52 nM and the IC_25_ was 3.86 nM; and the IC_50_ of H3122LR was 6.59 nM and the IC_25_ was 4.75 nM.

First, we detected the IC_50_ of bortezomib in 9 types of NSCLC cells (A549: 3.38 nM, H358: 3.12 nM, H1299: 3.1 nM, PC9: 7.45 nM, PC9OR: 8.72 nM, HCC827: 7.46 nM, HCC827OR: 8.13 nM, H3122: 4.52 nM and H3122LR: 6.59 nM) (Fig. S6A–I). It could be seen that the IC_50_ of bortezomib in all 9 types of NSCLC cells were less than 10 nM. Next, we examined the effect of bortezomib on the proliferation of NSCLC cells. We found that NSCLC cells in the bortezomib-treated groups had different degrees of rounding, brightening and decreased cell density compared with the control groups (Fig. 5A). Meanwhile, compared with the control groups, the rate of cell growth was slower (*P* < 0.0001, Fig. 5B), the number of cell clones was reduced (Fig. 5C), the cell cycle was arrested in the G0/G1 phase (Fig. 5D, Fig. S7A and Table S6), and the cell apoptosis was increased (Fig. S7B, C and Table S7) in the bortezomib-treated groups of 7 types of NSCLC cells.

In order to clarify the role of bortezomib in the NSCLC, we first detected the functional status of STAT3, PI3K-AKT, NF-κB, MAPK-p38 and MAPK-ERK1/2 signaling pathways after treated with IC_25_ and IC_50_ of bortezomib in the 7 types of NSCLC cells. We found that there was no apparent activation or inhibition of phosphorylated proteins in all 7 types of NSCLC cells, and only a few cells were inhibited in the same signaling pathways. For example, with increasing dosage of bortezomib, in A549 and H358 cells, the expression of p-STAT3 was decreased; in A549, H358 and PC9OR cells, the expression of p-NF-κB was decreased; in H1299, H358 and H3122 cells, the expression of p-p38 MAPK was decreased; and in A549, H358, PC9 and PC9OR cells, the expression of p-ERK1/2 was decreased (Fig. S8A, B). Next, we examined the effect of bortezomib on cell cycle pathway in NSCLC cells. As shown in the Fig. 5E, F of 7 types of NSCLC cells, with increasing dosage of bortezomib, the expressions of cell cycle blocking protein p16, p21 and p27 were significantly increased, and the expressions of CDK4, CDK6, CCND1 and CCNE1 involved in G1 phase were significantly decreased. In addition, the expressions of CDK2 and CCNA2 involved in S phase, CDK1 and CCNB1 involved in G2/M phase, and p57 involved in the entire cell cycle, had no significantly changed in 7 types of NSCLC cells (Fig. S8C, D).

### Bortezomib inhibits tumor growth of NSCLC in xenograft mice model

**Fig. 6:**
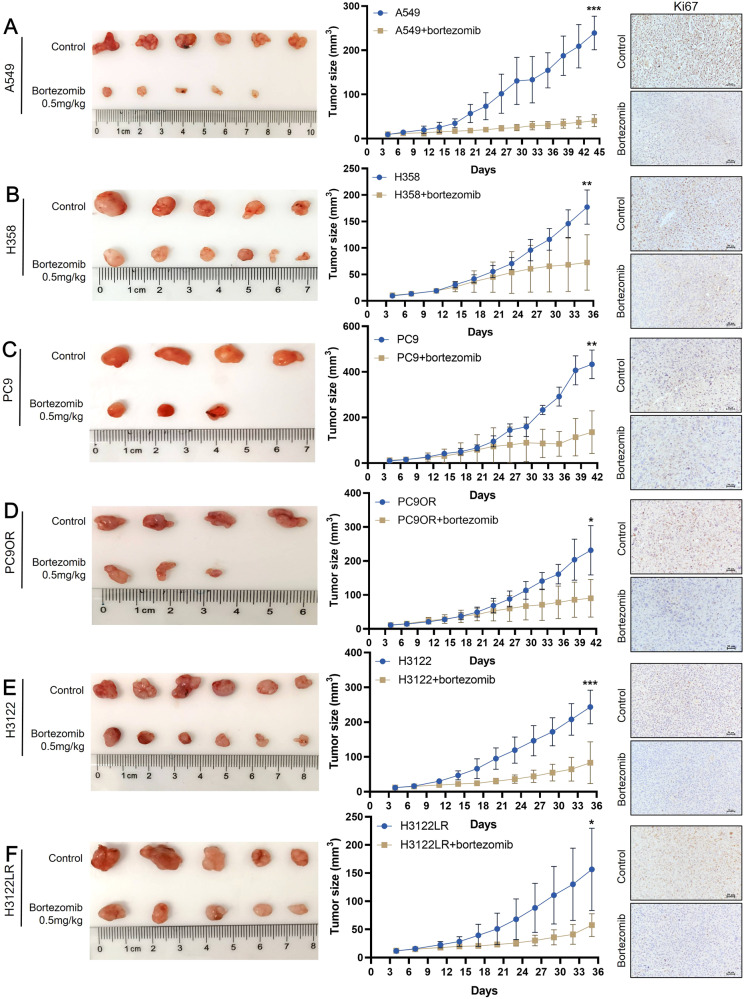
Bortezomib inhibits tumor growth in mice with NSCLC xenografts. **A**–**F** Tumor photographs, growth curves and the staining of Ki67 in six types of NSCLC xenograft mice (A549, H358, PC9, PC9OR, H3122 and H3122LR) after treated with bortezomib. *P < 0.05, **P < 0.01, ***P < 0.001.

In order to verify the result of bortezomib could inhibit the proliferation of NSCLC cells, we constructed subcutaneous xenograft models with 6 types of NSCLC cells (A549, H358, PC9, PC9OR, H3122 and H3122LR) and treated with bortezomib. Figure 6 shows the anatomical appearance, tumor growth curves and Ki67 staining in 6 xenograft mice models. Due to the individual difference of nude mice, some mice failed to form the subcutaneous tumors eventually. Therefore, we only showed the nude mice which had the subcutaneous tumor in this study (nude mice ≥3 in per group). Our results showed that in the bortezomib-treated groups, the tumor volume were significantly smaller and the staining intensity of Ki67 was weaker than that in the control groups (*P* < 0.05, Fig. 6A–F). At the same time, we stained cell cycle pathway-related molecules in tumor tissues, and the results showed that compared with the control groups, the staining of p21 and p27 was stronger, while the staining of CDK4 and CDK6 was weaker in the bortezomib-treated groups (Fig. S9A, B).

**Fig. 7:**
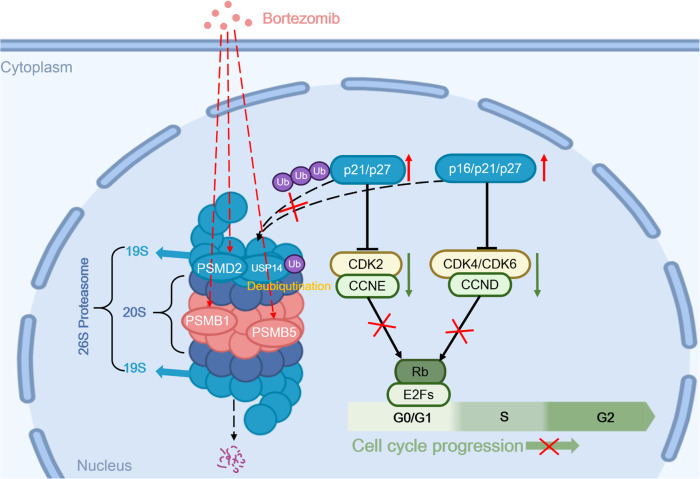
Bortezomib targets to proteasome subunit coding genes to inhibit cell proliferation of NSCLC. Under the synergistic effect of USP14, knocking out PSMD2 or treating with bortezomib can inhibit the degradation of proteasomes of p21 and p27, reduce the activity of cyclin/CDK complexes, and block the cell cycle in G0/G1 phase, thereby inhibiting the proliferation of NSCLC.

Combined with these results, we believe that by targeting proteasome coding genes of cell cycle check points, bortezomib could block the proteasome degradation of p21 and p27, hinder the formation of cyclin/CDK complex, arrest the cell cycle in G0/G1 phase, and then inhibit the proliferation of NSCLC cells (Fig. 7).

## Discussion

TKI treatment has become the preferred choice for NSCLC patients due to its specificity and mild adverse reactions, but the inevitable drug resistance significantly limits the long-term survival benefit of patients [32–35]. Meanwhile, there are still a large number of patients without sensitive driven mutations who are not available for TKI treatments [36]. Therefore, it is particularly important to find new therapeutic targets for NSCLC patients with different driven mutations or with TKI resistance. In recent years, network analysis has been widely used in study of various diseases, especially in the molecular characterization and key molecule identification of cancers [37–39]. Unlike traditional differential analysis, network analysis could isolated the potential important regulators in nodes via molecular network constructing, which is more efficiency and accurate [40]. In this study, we constructed a transcriptome network to discover new therapeutic target for NSCLC with different driven mutations or TKI resistance, and showed proteasome subunit coding genes were located in the central of cell cycle regulation network, which also suggested targeting proteasome subunit coding genes might be the common potential strategy for NSCLC.

Among these proteasome subunit coding genes, PSMD2 was extremely correlated with the prognosis of NSCLC patients, and could serve as independent prognostic factor. Studies have reported that PSMD2 is a non-ATP subunit of the 19S proteasome complex and is highly expressed and correlated with tumor stage in NSCLC [41]. In our study, we found that NSCLC patients with high expression of PSMD2 had poor prognosis (Fig. 2H). Consistent with this result, a prognosis prediction model consisting of 29 genes including PSMD2 was also associated with poor prognosis of patients in breast cancer [42]. Further exploring the role of PSMD2 in the progression of NSCLC, we found that the growth rate of NSCLC cells was significantly slower after knocked out PSMD2 (Fig. 3B, C). This result was also consistent with two previous studies about PSMD2: silencing PSMD2 can reduce the cell proliferation and induce apoptosis in NSCLC cells [41]; transfection of PSMD2 cDNA can stimulate the growth of hepatoma cell line SMMC-7721 and mouse embryonic fibroblast NIH-3T3 [43]. Previous studies have reported that PSMD2 co-localizes with p21 and p27 in the nucleus, and PSMD2 can mediate the ubiquitin-proteasome degradation of p21 and p27 under the collaboration with the USP14 [44, 45]. Our results showed that the protein expression of p21 and p27 was increased after knocked out PSMD2 (Fig. 3H), suggesting that knockout PSMD2 could block the ubiquitin-proteasome degradation of p21 and p27 and inhibit the proliferation of NSCLC cells.

As we all known, uncontrolled cyclin-dependent kinase activation is the cause of malignance, and their function is tightly regulated by cell cycle inhibitors such as p21 and p27 proteins. Following anti-mitotic signaling or DNA damage, p21 and p27 bind to the cyclin-CDK complex results in catalytic activity inhibition and cell cycle arrest [46]. Therefore, targeting the cell cycle regulation has been regarded as a promising anticancer strategy. The currently developed CDK4/6 inhibitors, such as ribociclib, oalbociclib and abemaciclib, are mainly used to treat ER+/HER2− breast cancer and can significantly prolong the PFS of patients [47]. In NSCLC, palbociclib and abemaciclib also obtain positive therapeutic effects [48, 49]. However, the adverse reactions (such as neutropenia, diarrhea, liver and kidney damage) and off-target effects of CDK4/6 inhibitors still limit the clinical benefits in patients [50]. Our data showed that bortezomib could also serve as an effective cell cycle inhibitor for NSCLC with different driven mutations (KRAS G12C/V mutations, EGFR mutation and ALK fusion) and TKIs resistance (osimertinib resistance and lorlatinib resistance).

Currently, the reported protein targets of bortezomib are PSMB1 and PSMB5 [30, 31]. In our research, we identified bortezomib could target PSMD2 (Fig. 4). Bortezomib, as the first proteasome inhibitor approved for clinical trial research, was used in the treatment of multiple myeloma [51, 52], and its role in the treatment of other hematological malignancies and solid tumors, such as indolent non Hodgkin’s lymphoma, mantle cell lymphoma, prostate cancer and lung cancer [53–55]. In this study, we demonstrated that compared with control group, after treated with bortezomib, the cell cycle was arrested in the G0/G1 phase (Fig. 5D), the expression of p21and p27 was increased (Fig. 5F), and the tumor volume in xenograft models was smaller (Fig. 6A–F). Take the above results together, we implied that bortezomib could inhibit the proteasome degradation of p21 and p27 by targeting proteasome subunit PSMD2, PSMB1 and PSMB5, to induce cell cycle arrest and inhibit the proliferation of NSCLC cells. However, it has been reported that bortezomib has some side effects when used alone to treat NSCLC, such as gastrointestinal side effects, increased dehydration rate, peripheral neuropathy and thrombocytopenia, which may be related to a higher initial dose or average dose density of bortezomib [56]. Therefore, exploring the dosage of bortezomib in vivo and preventing adverse reactions through combination therapy will be the key to our further research.

In conclusion, by introducing transcriptome network analysis into the discovery of NSCLC therapeutic target, we successfully identified PSMD2, which was significantly correlated with the poor prognosis of NSCLC patients and could serve as a novel therapeutic target. Bortezomib could target PSMD2, PSMB1 and PSMB5 to inhibit the proteasome degradation of cell cycle check points, to block cell proliferation and tumor growth of NSCLC, which was potential optional drug for NSCLC patients with different driven mutations or with TKI resistance.

## Materials and methods

### Isolation of transcriptome data and tissue microarray

RNA sequencing was performed on NSCLC cell lines, including H3122, H3122LR (Lorlatinib-resistant), PC9 and PC9OR (Osimertinib-resistant). Meanwhile, we downloaded the sequence data of A459, H1299, H358 and BEAS-2B (normal lung bronchial epithelial cells) from the GSE72794, GSE77209 and GSE172222 (http://www.ncbi.nlm.nih.gov/geo) [57]. The common genes were taken from all sequence data, and finally an expression matrix consisting of 9639 genes and 23 cell samples was obtained for subsequent analysis.

A total of 180-spot NSCLC tissue microarray composed of 98 NSCLC and 82 adjacent cancer samples were obtained from Shanghai Outdo Biotech CO., Ltd. This study was approved by the Ethics Committee of Shanghai Outdo Biotech CO., Ltd, with the ethics approval number YBM-05-02 and the informed consent was obtained from all subjects. All patients underwent surgery between January 2008 and July 2013 and were followed up for 3–8.5 years. All patients had no special medical history and did not receive preoperative chemotherapy and radiotherapy. The no special medical history refers to (within 5 years): no other history of malignant tumors; no history of pulmonary fibrosis, interstitial pneumonia, pneumoconiosis, radiation pneumonia, drug-related pneumonia, severe damage to lung function, and other diseases; no history of infectious diseases; no history of immune deficiency; no history of organ transplantation. During subsequent data analysis, 3 NSCLC samples with incomplete clinical information were removed, and 95 NSCLC samples remained.

### Data preprocessing and difference analysis

For the expression matrix mentioned above, the “removeBatchEffect” function of the “limma” package [58] was used to remove batch effects. After batch correction, 25% of genes with low average expression were removed, and then 25% of genes with large variance were removed [59], and finally 5421 genes were obtained for further analysis.

The corrected matrix contains 5421 genes and 23 cell samples. According to NSCLC and normal cell groupings, difference analysis was performed by the “limma” package. In order to retain all differential genes, only screened by *P* < 0.05, regardless of the logFC (fold change) (logFC = 0).

### Correlation network analysis and screening of functional cluster

We used spearman correlation analysis [60] to calculate the correlation coefficient matrix based on the differential expression genes. And according to the correlation coefficient >0.95 and degree >3, we got 1299 genes and 7343 gene pairs.

Then, we screened the functional clusters on the correlation network by the “MCODE” plugin of Cytoscape (3.7.2) [61, 62]. According to degree cutoff =2, node score cutoff =0.2, K-Core =2 and score >10, 10 clusters were obtained. Finally, we plotted this correlation network through Cytoscape and the biological functions of different clusters were enriched by “clusterProfiler” R package [63].

### Causality network analysis

We used the “bnlearn” R package [64] to perform a Bayesian causality analysis on the above-selected cluster_2 and cluster_9. The causality networks of cluster_2 and cluster_9 were drawn by Cytoscape (3.7.2), and the candidate genes were obtained by weight >0.8 and out-degree ≥2.

### Survival analysis

Through univariate and multivariate Cox regression analysis, we identified PSMD2 was the prognosis-related gene in NSCLC. The “survival” and “survminer” R packages were used for survival analysis. The Kaplan-Meier method was used to estimate the survival curve, and the log-rank test was used to analyze the difference in survival time.

### Functional enrichment analysis

Based on the protein–protein interaction (PPI) analysis, we identified 269 PSMD2-related proteins. These proteins were subjected to GO terms enrichment analysis using “clusterProfiler” R package [63]. Lung adenocarcinoma (LUAD) samples of TCGA database were divided into two groups (high and low) based on the expression of PSMD2, GSEA (http://software.broadinstitute.org/gsea/index.jsp) [65] was performed between the two groups.

### Thermal shift assay

Experimental procedures for thermal shift assay have been reported [66]. (1) Prepare four culture dishes (10 cm) filled with NSCLC cells, two for the treatment group and two for the control group. The treatment group was treated with tenfold IC_50_ of bortezomib, while the control group was incubated with corresponding amounts of DMSO at 37 °C in a 5% CO_2_ incubator for 1 h. (2) Discard the supernatant, digest the cells with trypsin into a 15 ml tube, centrifuge 300 × *g* for 3 min; Discard the supernatant and resuspend the cell precipitate with pre cooled 5 ml PBS solution, centrifuge 300 × *g* for 3 min; Discard the supernatant and resuspend the cell precipitate with 1.5 ml pre cooled PBS containing 1% protease inhibitor, transfer to a new 2 ml EP tube. (3) Take 100 μl of cell suspension from each treatment group and control group into a 200 μl PCR tube, ensuring that every 100 μl contains 3 × 10^6^ cells. Set the temperature range between 50 °C and 70 °C using a PCR instrument, with 14 types of temperature wells. Heat the sample for 3 min, then place the PCR tube at room temperature for 3 min, and then transfer it to liquid nitrogen for quick freezing. (4) Repeat freeze-thaw twice on the cell suspension using liquid nitrogen and a 25 °C constant temperature metal bath. After each thaw, a vortex is required, and finally the cell lysate is placed on ice. (5) Transfer the cell mixture from all PCR tubes to a new 1.5 ml EP tube, centrifuge 17,000 × *g* at 4 °C for 20 min, and collect 90 μl of supernatant into the new 1.5 ml EP tube on ice. (6) BCA protein assay kit (Solarbio, Beijing, China) detects protein concentration, and then adds 5× loading buffer to heat in a 100 °C metal bath for 10 min for subsequent experiments.

### Molecular docking

The SDF file of bortezomib structure was downloaded from the PubChem database [67]. The PDB files of the crystal structures of PSMD2 (PDB ID: 5GJQ), PSMB1 (PDB ID: 4R3O), PSMB2 (PDB ID: 4R67) and PSMB5 (PDB ID: 5L5W) were downloaded from the RCSB Protein Data Bank (PDB) database [68]. Ligands and receptors were pretreated and prepared according to the tutorial and manual of AutoDock Tools (http://vina.scripps.edu/manual.html) [69]. Bortezomib docks with the corresponding protein receptor via AutoDock Vina, and compound-target pairs with docking fraction less than −5 kcal/mol are considered as binding pairs [70]. The PLIP platform was used to analyze the binding sites of compound-target pairs [71]. PyMol was used to visualize the results of AutoDock Vina and PLIP [72].

### Cell culture, reagents, and lentivirus infection

NSCLC cell lines (A549, H1299, H358, H3122, HCC827 and PC9) and normal lung bronchial epithelial cells BEAS-2B were purchased from Shanghai Academy of Science (Shanghai, China). H358, A549, H1299, HCC827 and H3122 cells were maintained in RPMI-1640 medium (Gibco, USA). BEAS-2B and PC9 were maintained in DMEM medium (Gibco, USA). All media were supplemented with 1% penicillin and streptomycin (Gibco, USA) and 10% fetal bovine serum (Gimini, USA). The dose of bortezomib (PS-341) (#S1013, Selleck Chemicals, Co., Ltd, USA) in NSCLC cells was mentioned below.

Briefly, knockout of PSMD2 was performed using sgPSMD2 (PSMD2-F:caccgCAAGTATCGGCTAGTGGGCT, R:aaacAGCCCACTAGCCGATACTTGc) and lentiCRISPRv2 plasmids (Zhang Lab) compared to a negative control of sgNC encoding a nonspecific 20nt guide RNA. The cells were co-transfected with lentiCRISPRv2 plasmids with CRISPR/Cas9 sgRNA. Two days after the transfection, cells were selected by puromycin for 1 week to obtain stable cell lines.

### Cell proliferation assay

Cells in logarithmic growth phase were taken and seeded into 96-well plates at 3000 cells per well. Five parallel control wells were set for each group of cells, and 4, 24, 48, 72 and 96 h were set as detection time points. After adding CCK-8 (CCK-8; Beyotime, Shanghai, China), the 96-well plate was placed in a constant temperature incubator for 1 h. Finally, use the microplate spectrophotometer (BioTEk, VT, USA) to detect the OD value at a wavelength of 450 nm, and calculate the relative cell growth rate at each time point.

### Western blot analysis

NSCLC cells were lysed in RIPA buffer (#R0100, Solarbio, Beijing, China) with protease and PMSF inhibitor cocktails. Protein concentration was determined using BCA protein assay kit (#PC0020, Solarbio). An equal amount of proteins (30 µg) for each sample were loaded on 10% SDS-polyacrylamide gels and then transferred to PVDF membranes (Merck Millipore, Cork, IRL). Membranes were subsequently blocked with TBST containing 5% skim milk at room temperature for 1 h and incubated with diluted primary antibody at 4 °C overnight. The next day, incubated the secondary antibody and exposed the bands with ECL luminescent solution (#PE0010, Solarbio). Primary antibodies used were as follows: Rabbit anti-PSMD2 (#14748-1-AP, Proteintech, Wuhan, China), Rabbit anti-CDK1 (#19532-1-AP, Proteintech), Rabbit anti-p21 (#10355-1-AP, Proteintech), Rabbit anti-p27 (#25614-1-AP, Proteintech), Rabbit anti-p16 (#10883-1-AP, Proteintech), Rabbit anti-p57 (#23317-1-AP, Proteintech), Rabbit anti-CDK2(#10122-1-AP, Proteintech), Rabbit anti-CDK4 (#11026-1-AP, Proteintech), Rabbit anti-CDK6 (#14052-1-AP, Proteintech), Rabbit anti-Ki67 (#27309-1-AP, Proteintech), Rabbit anti-p-IκBα (#2859T, Cell Signaling Technology, Massachusetts, USA), Rabbit anti-IκBα(#4814T, CST), Rabbit anti-p-NF-κB p65(#8242T, CST), Rabbit anti-NF-κB p65 (#3033T, CST), Rabbit anti-p-ERK1/2 (#310065, ZEN BIO), Rabbit anti-ERK1/2 (#343830, ZEN BIO), Rabbit anti-STAT3 (#10253-1-AP, Proteintech), Rabbit anti-p-STAT3 (#9145S, CST), Rabbit anti-p44/42 MAPK (#4695T, CST), Rabbit anti-p-p44/42 MAPK (#4370T, CST), Rabbit anti-AKT (#51077-1-AP, Proteintech), Mouse anti-p-AKT (#66444-1-Ig, Proteintech), Rabbit anti-cyclin D1 (#60186-1-AP, Proteintech), Rabbit anti-cyclin A2 (#18202-1-AP, Proteintech), Rabbit anti-cyclin B1 (#55004-1-AP, Proteintech), Rabbit anti-cyclin E1 (#11554-1-AP, Proteintech), Rabbit anti-PSMB1 (#11749-1-AP, Proteintech), Rabbit anti-PSMB5 (#19178-1-AP, Proteintech), Mouse anti-GAPDH (#1E6D9, Proteintech) and Mouse anti-β-actin (#66009-1-Ig, Proteintech).

### Colony formation assay

The NSCLC cells were seeded in 6-well plates (500 cells per well) treated with bortezomib in the next day with culture medium for 2 weeks. The cells were washed twice with PBS and fixed with 4% paraformaldehyde for 30 min followed by 0.1% crystal violet staining for 15 min. Afterwards, the cells were re-washed twice with PBS and imaged by a digital camera.

### Cell cycle and apoptosis assays

Cells were harvested when NSCLC cells reached 90% confluence. After fixation with 70% ethanol at 4 °C overnight, cells were re-washed twice, and then stained with propidium iodide (PI)/RNase Staining Solution (#C1052, Beyotime, Shanghai, China) for 30 min in the dark. The proportion of cell cycle phases was detected by flow cytometry (Cytoflex, Beckman, Germany).

For apoptosis assay, cells were treated with Annexin V-FITC Apoptosis Detection Kit (#C1052, Beyotime) and determined by cell sorting. Approximately 20,000 cells were collected per experiment and assays were repeated three times independently.

### Immunohistochemistry (IHC) and quantification

Formalin-fixed and paraffin-embedded sections were baked at 65 °C for 4 h, then deparaffinized, hydrated, and subjected to antigen retrieval. Sections were incubated with Rabbit anti-PSMD2 (1:200; #14781-1-AP, Proteintech), Rabbit anti-p21 (1:200; #10355-1-AP, Proteintech), Rabbit anti-p27 (1:200; #25614-1-AP, Proteintech), Rabbit anti-CDK4 (1:200; #11026-1-AP, Proteintech) and Rabbit anti-CDK6 (1:200; #14052-1-AP, Proteintech) overnight at 4 °C. Subsequently, these tissue sections were incubated with horseradish peroxidase-conjugated secondary antibody (1:1000, ZSGB-BIO) for 5 min at 37 °C. Then, sections were stained with DAB+ substrate chromogen solution (#ZLI-9017, ZSGB-BIO, Beijing, China) for 1 min at room temperature, followed by counterstaining with hematoxylin. Two researchers scored the staining results in a blinded fashion independently.

The protein expression was evaluated by both staining intensity and percentage of staining positive cells based on a semi-quantitative scoring system [73]. Staining intensity was defined as 0 for negative staining, 1 for weak staining, 2 for moderate staining and 3 for strong staining. Percentage of positive cells was quantified as 0 for ≤5% positive cells, 1 for 6–25%, 2 for 26–50%, 3 for 51–75% and 4 for >75%. The sum of the staining intensity and the percentage of positive cells score was determined as the positive grade of the slides. A score of 0 represented negative, 1–3 (+) represented weakly positive, 4-5 (++) represented positive, and 6–7 (+++) represented strongly positive. In this study, patients were divided into two groups(-/+) based on the IHC score of PSMD2, with score ≤3 representing the “-” subgroup and score >3 representing the “+” subgroup.

### In vivo experiment

Female nude mice were purchased from GemPharmatech Co., Ltd (Jiangsu, China) and housed in facilities approved by Animal Care and Use Committee of West China Hospital, Sichuan University. Nude mice were housed in an SPF environment for 1 week, and then each mouse was subcutaneously injected with 100 μl of 5 × 10^6^ NSCLC cells. Tumor size was measured every 3 days (tumor size = *a* × *b*^2^/2). When the tumor size had reached 80–120 mm^3^, the mice were randomly divided into two groups (no blinding). Mice were injected subcutaneously twice a week (3 weeks) with 0.5 mg/kg bortezomib (#S1013, Selleck Chemicals, Co., Ltd, USA). Then, all mice were sacrificed on day 45, xenograft tumors were dissected and its size were measured. Finally, tumor tissues were fixed with 4% formalin, and the corresponding analysis was performed by immunohistochemistry.

### Data analysis

Statistical analysis of all experimental data was performed using SPSS 22.0 (SPSS Inc, Chicago, IL, USA) and graphical presentation of experimental results was performed using Graphpad Prism 8.0 (Graphpad Inc, La Jolla, CA, USA). Semi-quantitative results of all CCK-8 assays and western blot were analyzed using *t*-test or Mann–Whitney *U* test. The Wilcoxon test was used to compare the expression of PSMD2 in normal lung and tumor tissues. The chi-square test was used to analyze the correlation between the IHC score of PSMD2 and the clinical features of NSCLC patients. The effects of bortezomib on colony formation, cell cycle and apoptosis in NSCLC cells were analyzed using one-way ANOVA. All data with normal distribution and homogeneity of variance were expressed as mean ± standard deviation. All results were considered statistically significant at *P* < 0.05 (*P < 0.05, **P < 0.01, ***P < 0.001, ****P < 0.0001).

### Supplementary information

Supplementary Figure S1-S9

Table S1

Table S2

Table S3

Table S4

Table S5

Table S6

Table S7

## Data Availability

Source data and reagents are available from the corresponding author (Email: senyi_deng@scu.edu.cn) upon reasonable request.
