# Supplementary material for: Dual network analysis of transcriptome data for discovery of new therapeutic targets in non-small cell lung cancer
Source: Oncogene. 2023 Oct 20;42(49):3605–18. doi: 10.1038/s41388-023-02866-5 (PMC10691970; doi:10.1038/s41388-023-02866-5)
Supplement: Supplementary file 1 — Supplementary Figure S1-S9 [file 41388_2023_2866_MOESM1_ESM.docx]

**Supplementary Figures**

**
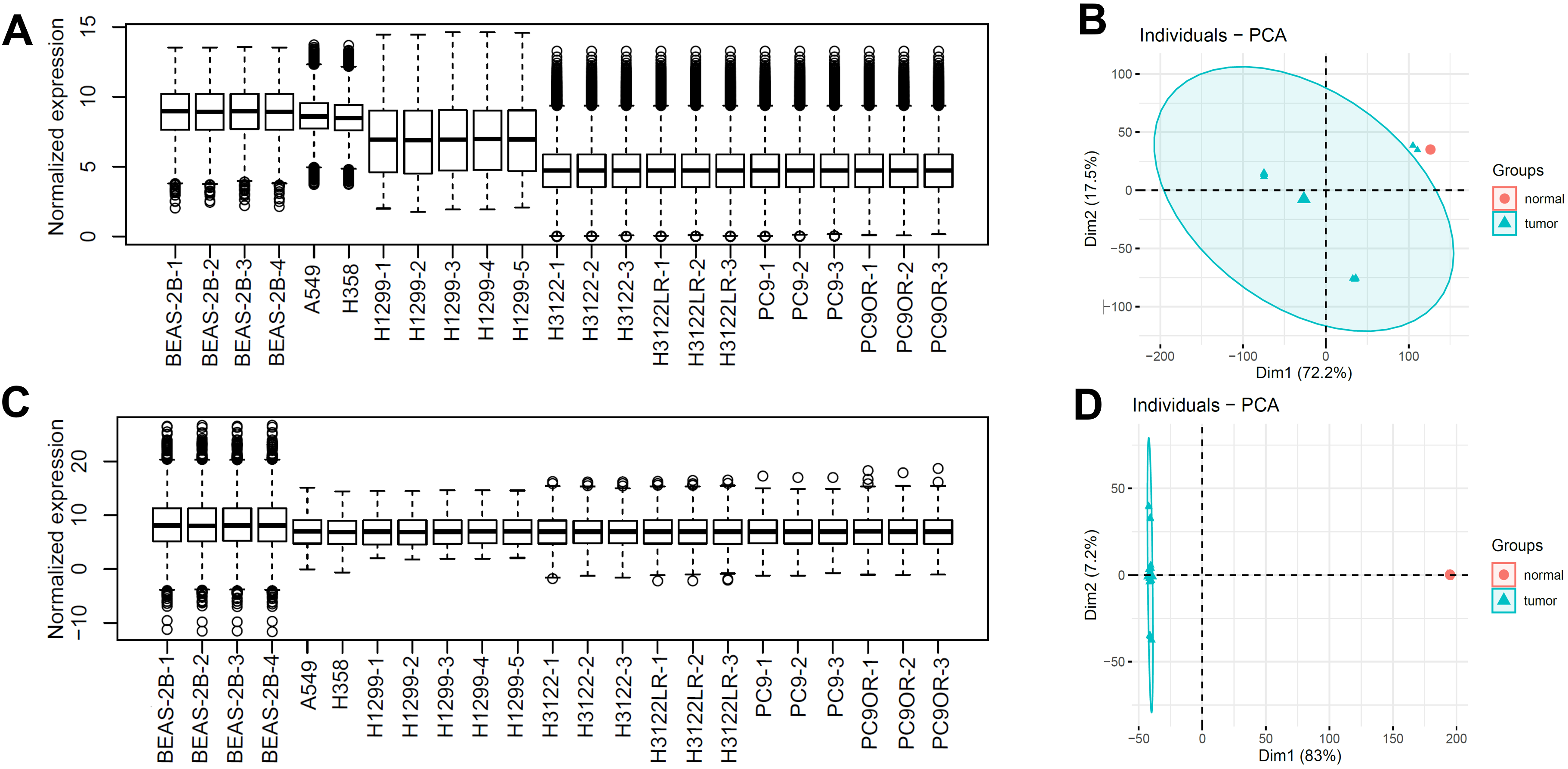
**

**Figure S1. Batch correction of seven NSCLC cell lines and BEAS-2B.** (A, B) Boxplot and PCA were shown the expression distribution of eight cell lines before batch correction. (C, D) Boxplot and PCA were shown the expression distribution of eight cell lines after batch correction.


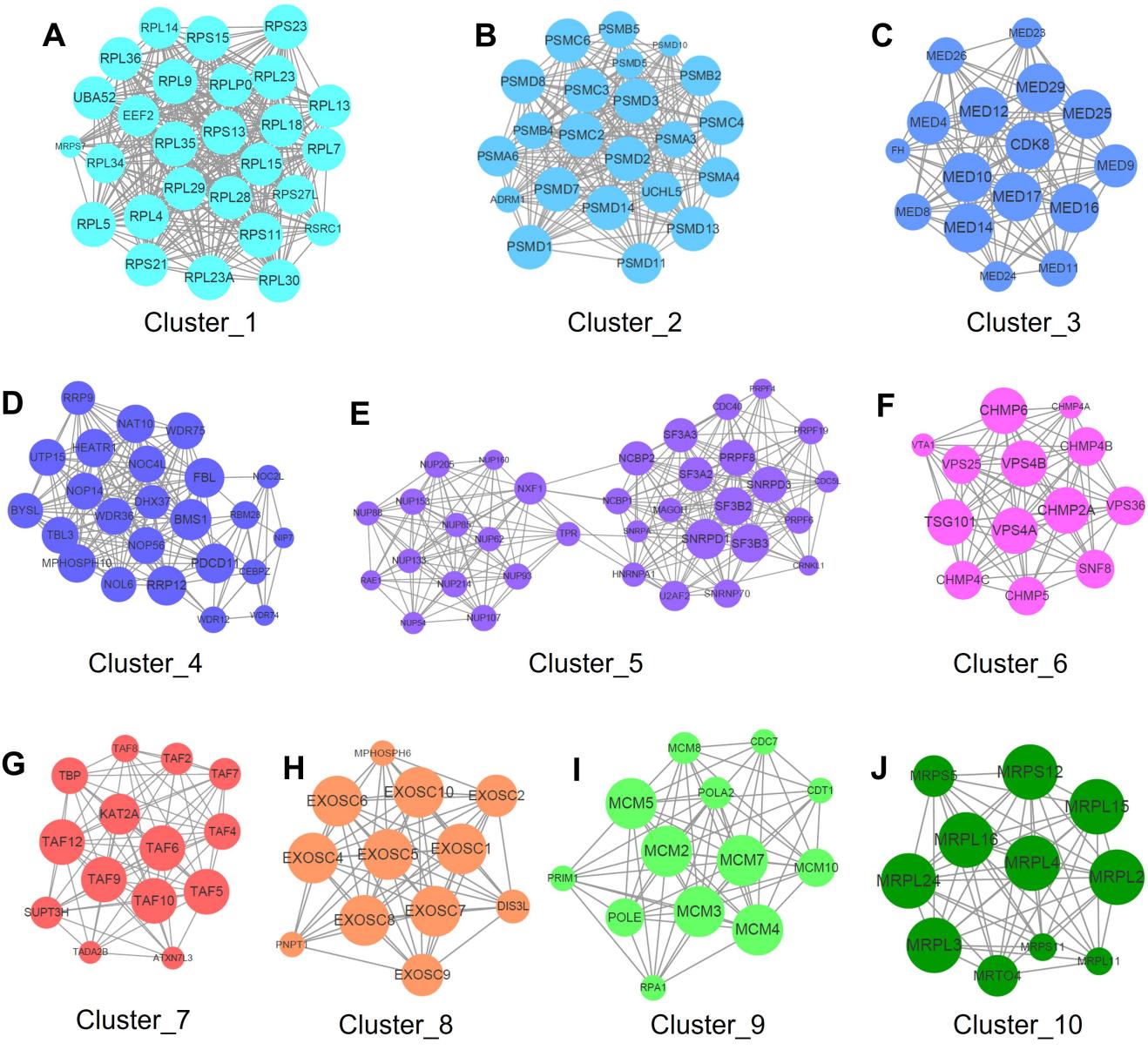


**Figure S2. Correlation network of ten functional clusters.** Different functional clusters were shown in different colors. The node size represents the degree size of genes.

**
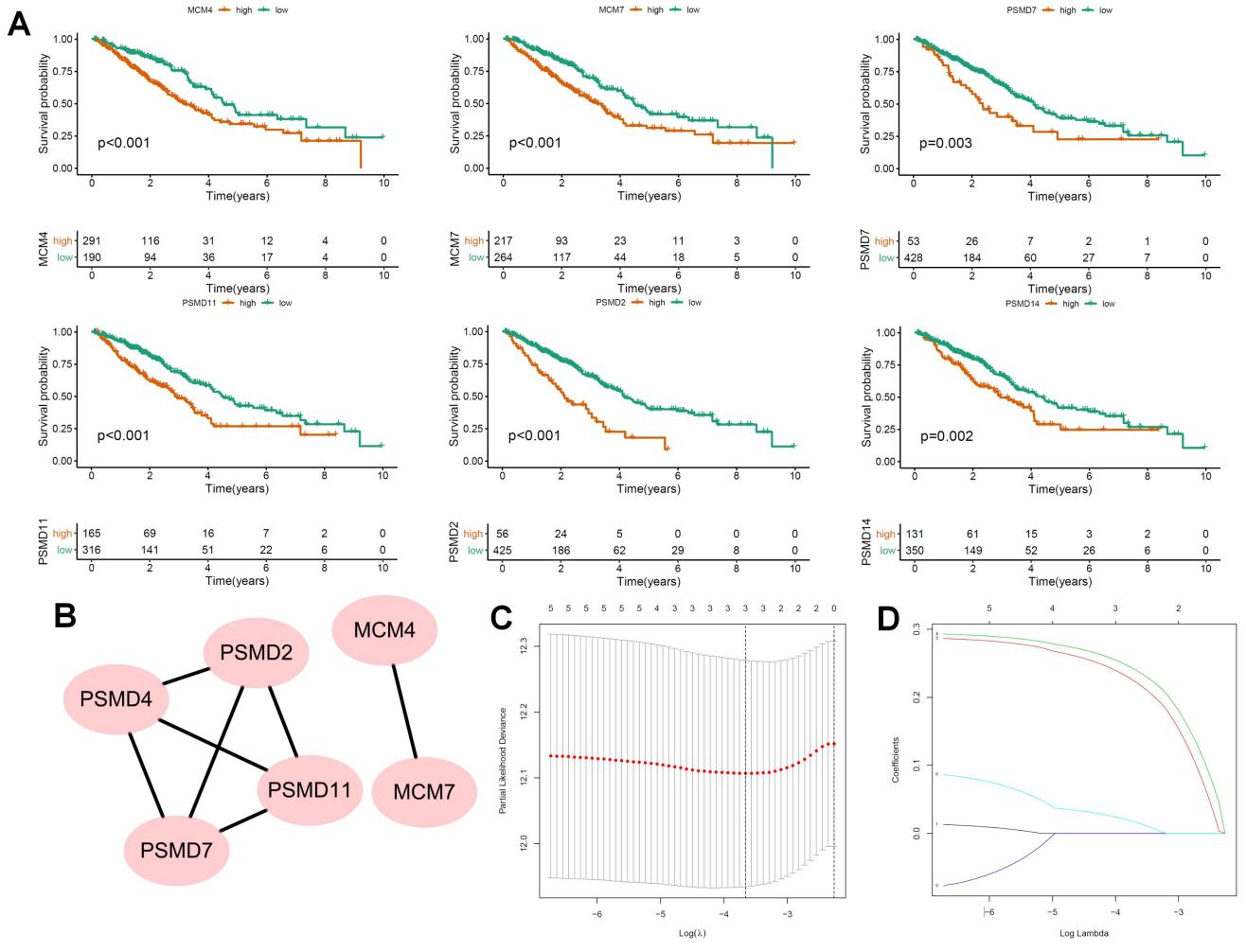
**

**Figure S3. PSMD2 was associated with poor prognosis in NSCLC patients and was enriched in cell cycle pathways.** (A) Survival curves of six prognosis-related genes (MCM4, MCM7, PSMD7, PSMD11, PSMD2 and PSMD14) in NSCLC patients. (B) STRING network analysis identifies the correlation network of six candidate genes. (C, D) Lasso regression analysis was used to determine the main feature genes.

**
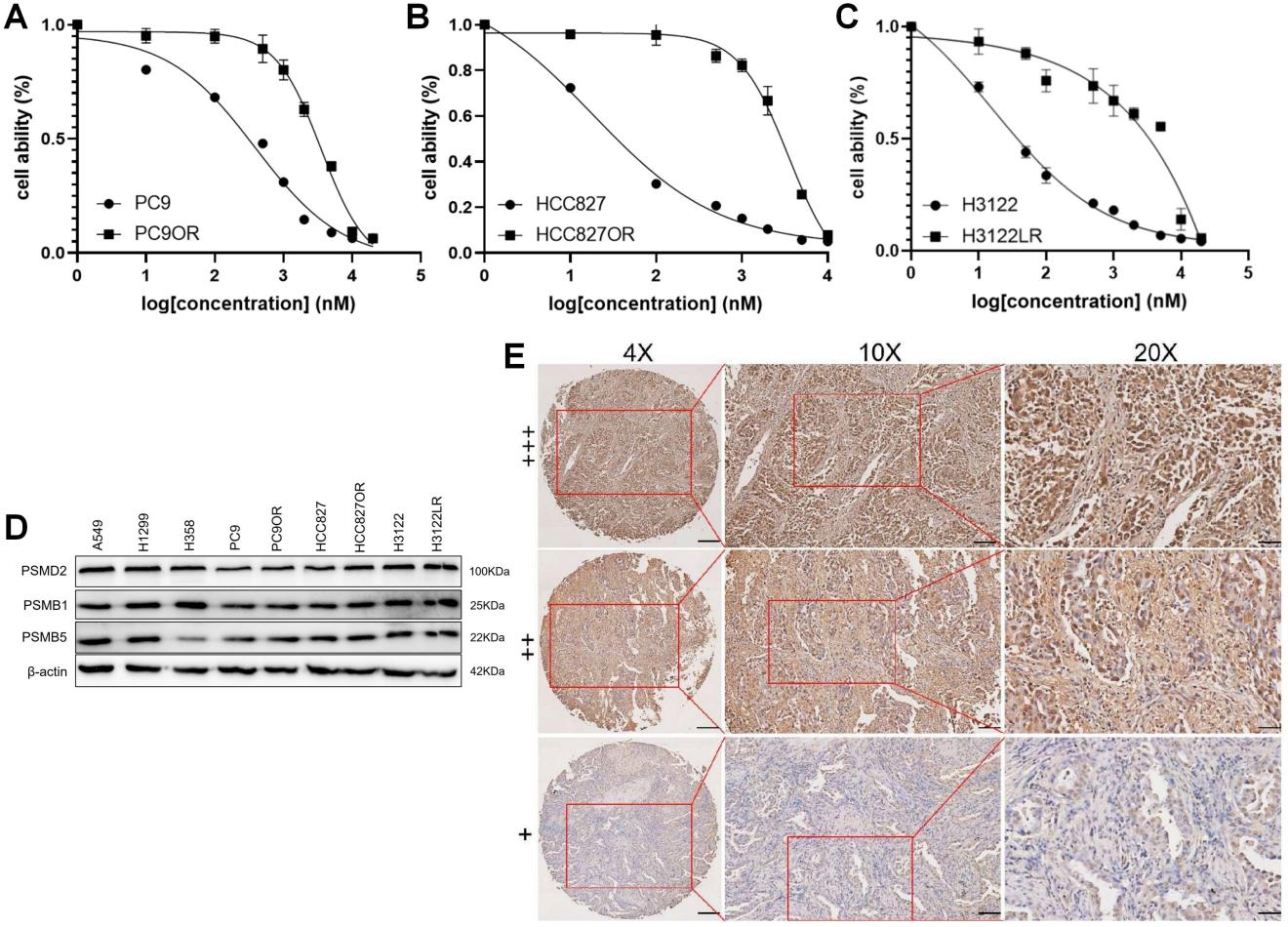
**

**Figure S4. PSMD2 was highly expressed in nine different types of NSCLC cells.** (A-C) IC50 of Osimertinib and Lorlatinib was detected by CCK-8 in PC9, PC9OR, HCC827, HCC827OR, H3122 and H3122LR. (D) The expression of PSMD2, PSMB1 and PSMB5 were detected in nine NSCLC cell lines. (E) Representative images of weak positive (+), positive (++), and strong positive (+++) of PSMD2 in tissue microarray.

**
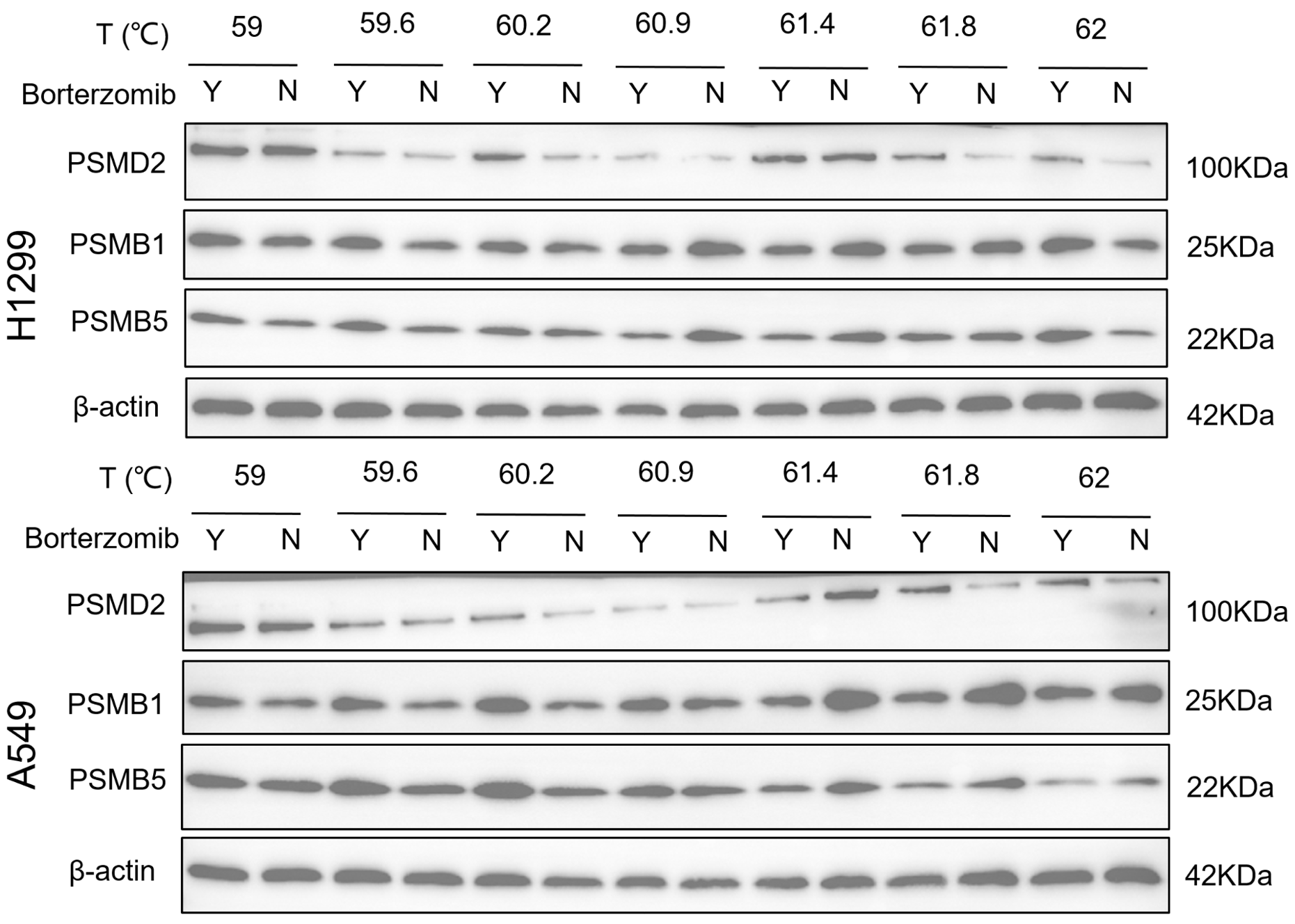
**

**Figure S5. Bortezomib could bind to PSMD2.** In H1299 and A549 cells, seven temperature gradients were set between 59°C and 62°C to detect the optimal temperature for bortezomib binding to PSMD2. Y represents cells were treated with bortezomib for one hour, and N represents treatment with an equal amount of DMSO for one hour.

**
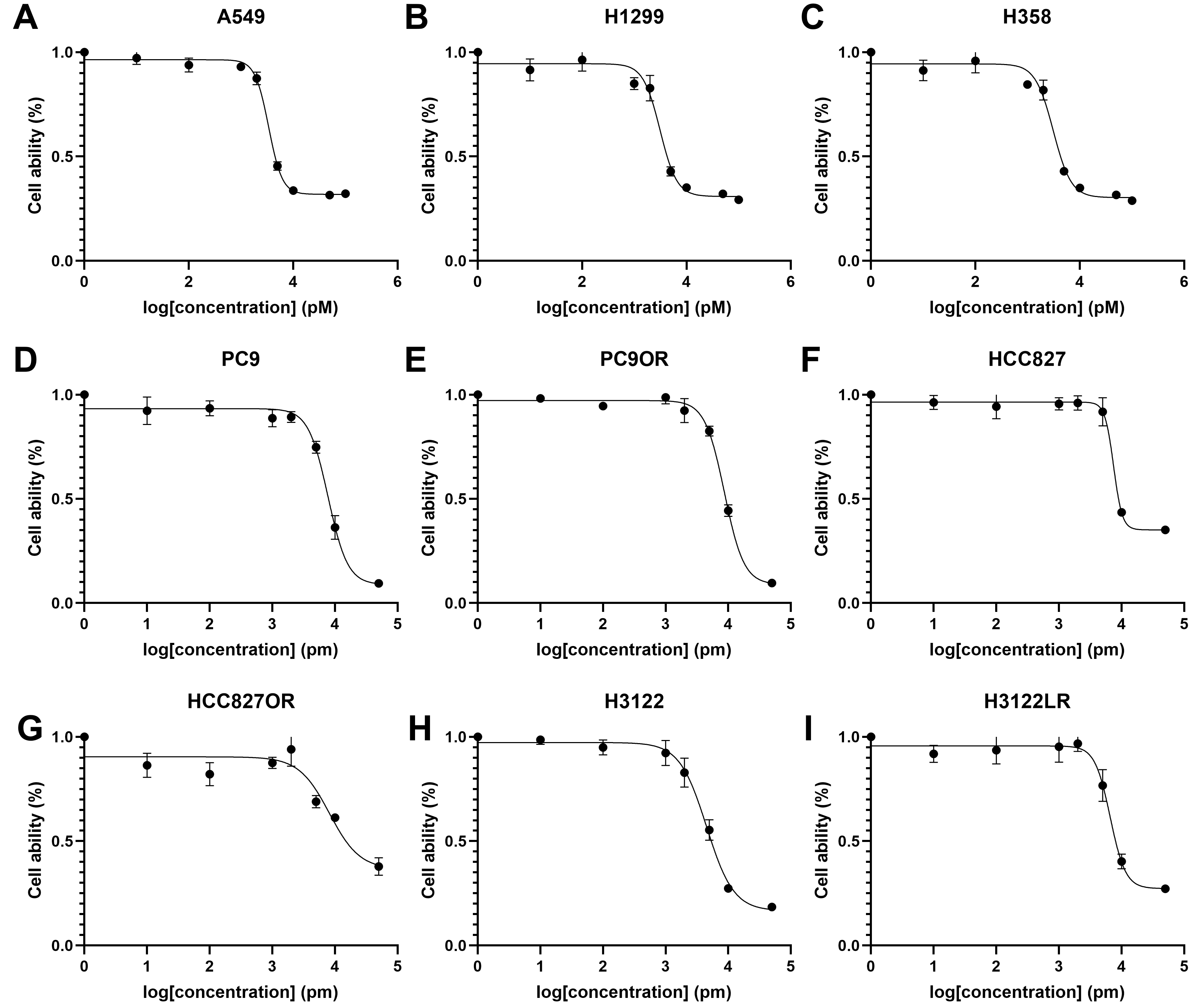
**

**Figure S6. The IC_50_ of bortezomib in nine different types of NSCLC cells was less than 10nM.** (A-I)The IC_50_ of bortezomib was detected by CCK-8 in A549, H1299, H358, PC9, PC9OR, HCC827, HCC827OR, H3122 and H3122LR cells.

**
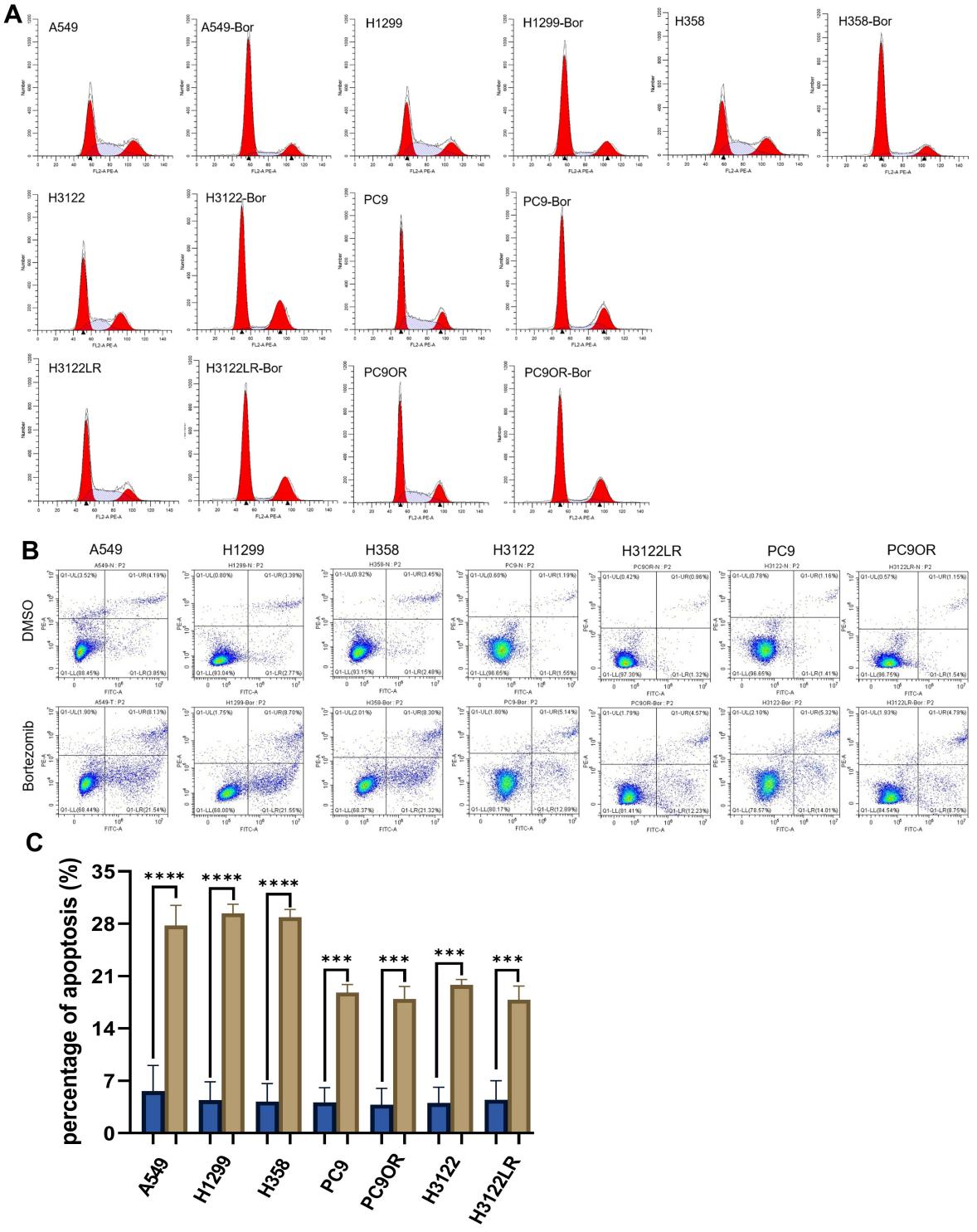
**

**Figure S7. Bortezomib could inhibit cell cycle and promote apoptosis in NSCLC cells.** (A) After treated with bortezomib, the percentages of seven types of NSCLC cells in different phase of cell cycle (G0/G1, S and G2/M) were detected by flow cytometry. (B, C) The cell apoptosis of seven types of NSCLC cells after bortezomib treatment for 48hours was detected by Annexin V/PI staining, and the statistical analysis was performed. ***P <0.001, ****P <0.0001.

**
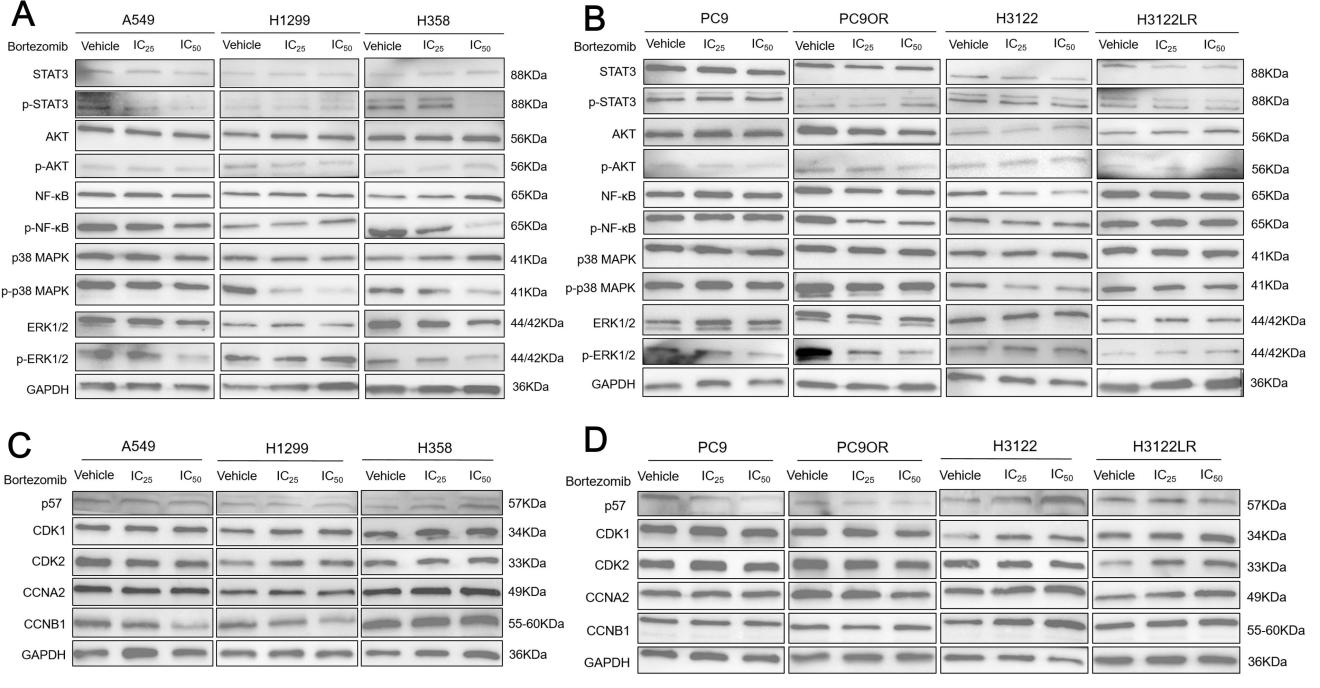
**

**Figure S8. Bortezomib did not inhibit the proliferation of NSCLC cells through cascade signaling pathway.** (A-B) The protein expression of cascade signaling pathway molecules in A549, H1299, H358, PC9, PC9OR, H3122 and H3122LR cells under different concentrations of bortezomib. (C-D) The protein expression of cell cycle pathway molecules in A549, H1299, H358, PC9, PC9OR, H3122 and H3122LR cells under different concentrations of bortezomib.

**
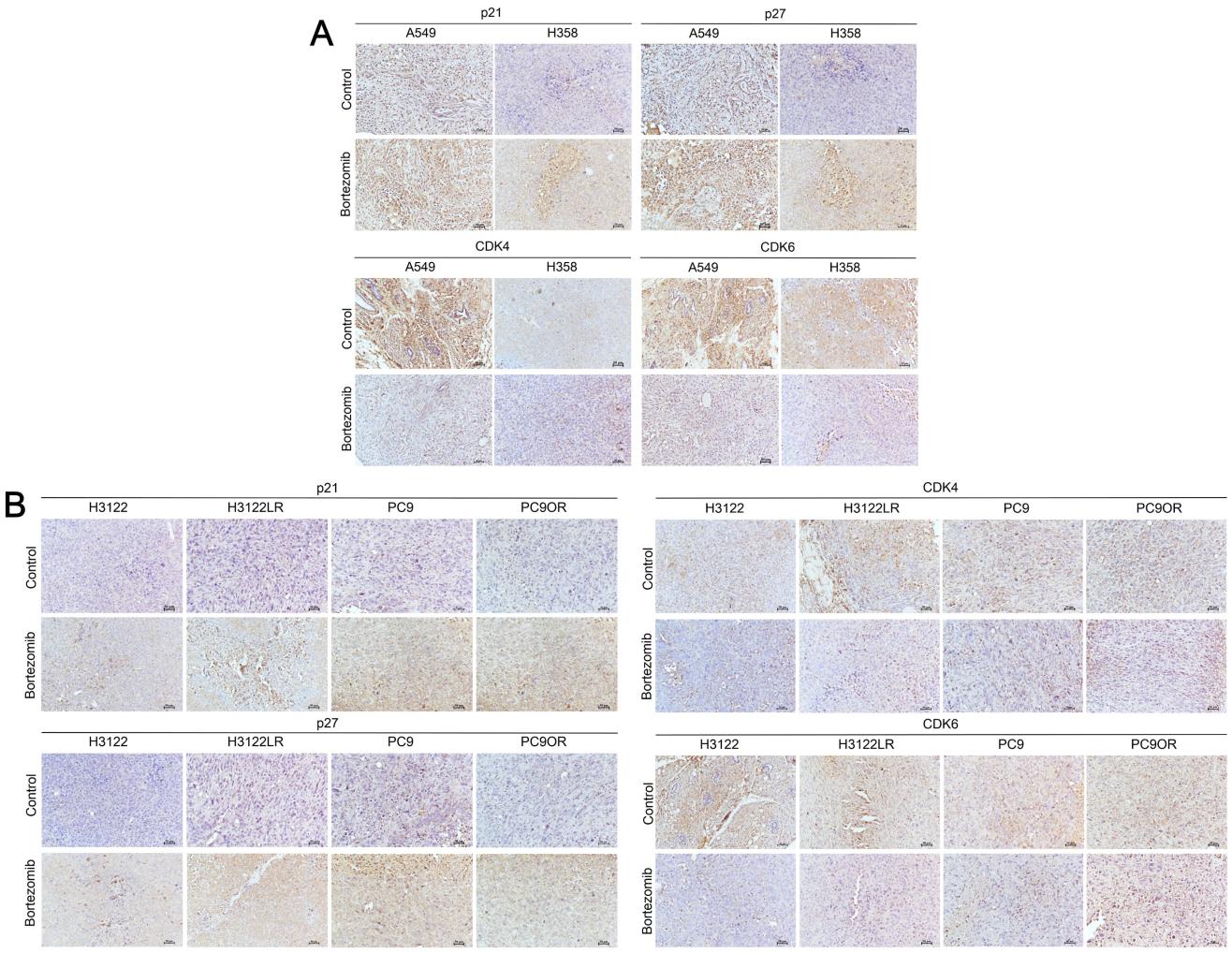
**

**Figure S9. After treated with bortezomib in xenograft mice, the staining intensity of p21 and p27 was increased, and the staining intensity of CDK4 and CDK6 was decreased.** (A, B) IHC staining of p21, p27, CDK4 and CDK6 in tumor tissues of six types of xenograft mice models of NSCLC cells.
